# Maternal diabetes modulates dental epithelial stem cells proliferation and self-renewal in offspring through apurinic/apyrimidinicendonuclease 1-mediated DNA methylation

**DOI:** 10.1038/srep40762

**Published:** 2017-01-17

**Authors:** Guoqing Chen, Jie Chen, Zhiling Yan, Ziyue Li, Mei Yu, Weihua Guo, Weidong Tian

**Affiliations:** 1 State Key Laboratory of Oral Diseases, West China College of Stomatology, Sichuan University, Chengdu, 610041, P. R. China; 2 National Engineering Laboratory for Oral Regenerative Medicine, West China College of Stomatology, Sichuan University, Chengdu, 610041, P. R. China; 3 Department of Oral and Maxillofacial Surgery, West China College of Stomatology, Sichuan University, Chengdu, 610041, P. R. China; 4 Department of Pedodontics, West China College of Stomatology, Sichuan University, No. 14, 3rd Section, Renmin South Road, Chengdu 610041, P. R. China

## Abstract

Maternal gestational diabetes mellitus (GDM) has many adverse effects on the development of offspring. Aberrant DNA methylation is a potential mechanism associated with these effects. However, the effects of GDM on tooth development and the underlying mechanisms have not been thoroughly investigated. In the present study, a GDM rat model was established and incisor labial cervical loop tissue and dental epithelial stem cells (DESCs) were harvested from neonates of diabetic and control dams. GDM significantly suppressed incisor enamel formation and DESCs proliferation and self-renewal in offspring. Gene expression profiles showed that *Apex1* was significantly downregulated in the offspring of diabetic dams. *In vitro*, gain and loss of function analyses showed that APEX1 was critical for DESCs proliferation and self-renewal and *Oct4* and *Nanog* regulation via promoter methylation. *In vivo*, we confirmed that GDM resulted in significant downregulation of *Oct4* and *Nanog* and hypermethylation of their promoters. Moreover, we found that APEX1 modulated DNA methylation by regulating DNMT1 expression through ERK and JNK signalling. In summary, our data suggest that GDM-induced APEX1 downregulation increased DNMT1 expression, thereby inhibiting *Oct4* and *Nanog* expression, through promoter hypermethylation, resulting in suppression of DESCs proliferation and self-renewal, as well as enamel formation.

Gestational diabetes mellitus (GDM) is the most common complication of pregnancy. The prevalence of GDM ranges from 2% to 6% and reaches up to 20% in specific high-risk populations^1^. A number of epidemiological studies have demonstrated that *in utero* exposure to maternal hyperglycaemia, induced by GDM, has detrimental effects on cardiovascular and urinary system development, and is linked to obesity and associated metabolic complications in the offspring^2^,^3^,^4^,^5^,^6^. Moreover, epidemiologic and animal model studies have shown that hyperglycaemia alters the tooth development process, affecting tooth eruption and mineralization^7^,^8^,^9^,^10^. However, the effects of maternal diabetes on tooth development, and the associated underlying mechanisms have not been thoroughly investigated.

The altered pregnancy environment associated with GDM is thought to be a risk factor for aberrant foetal development, through changes in developmental programming^11^,^12^. Accumulating evidence indicates that DNA methylation can serve as a bridge between environmental changes and cellular responses. During embryonic development, DNA methylation undergoes reprogramming and is particularly sensitive to the *in utero* environment^13^,^14^,^15^; moreover, the epigenetic signature acquired *in utero* might have long-term consequences for gene expression and could alter cellular and tissue phenotypes^16^,^17^,^18^. Currently, severe hyperglycaemia during pregnancy is thought to be one of the most important determinants of aberrant foetal development. However, whether maternal gestational diabetes modulates tooth morphogenesis in offspring through altered DNA methylation remains largely unknown.

Apurinic/apyrimidinicendonuclease 1 (APEX1) is a multifunctional mammalian protein that not only plays a central role in DNA base excision repair but also acts as a transcriptional regulator^19^,^20^. During embryonic development, APEX1 is critical for cell survival, and *Apex1*-null mice show early embryonic lethality^21^. APEX1 has also been implicated in the maintenance of stem cell pools through its modulation of intracellular redox homeostasis^22^. Our previous study confirmed the regulation of dental papilla cell differentiation by APEX1, through its redox function^23^. However, to date, there have been few reports on the function of APEX1 in tooth development and pathology.

Dental epithelial stem cells (DESCs), located in the labial cervical loop (LaCL), are known to give rise to transient cells that propagate, migrate anteriorly, and then differentiate into ameloblasts that produce enamel matrix^24^,^25^,^26^,^27^. The growth of incisors is supported by the division of DESCs, and modulation of DESCs might affect the growth of incisors and enamel formation. In the present study, we aimed to elucidate the underlying mechanisms associated with the reprogramming effects of a hyperglycaemic *in utero* environment, induced by maternal diabetes, on the DESCs of offspring. Our results showed that exposure to this environment has dramatic effects on proliferation and self-renewal in offspring DESCs, indicating critical APEX1-mediated dysregulation of DNA methylation, and providing novel insights into the mechanisms of DNA methylation-induced reprogramming of tooth development induced by maternal diabetes.

## Results

### Maternal diabetes impairs incisor enamel morphogenesis in offspring

**Figure 1:**
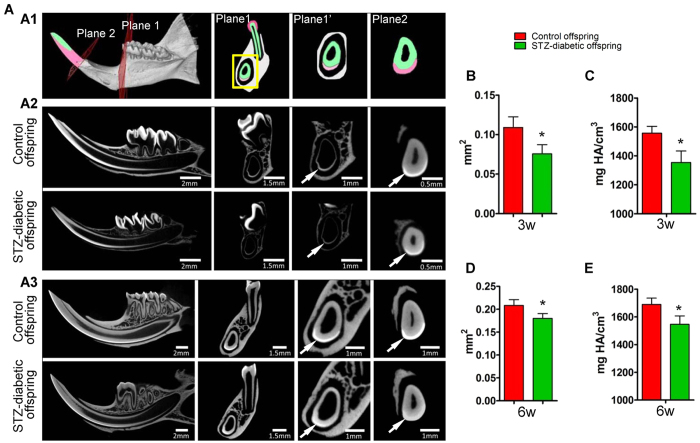
Maternal diabetes impairs enamel formation in offspring. (**A**) Micro-computed tomography (CT) analysis of incisors from the offspring of control and diabetic dams at three and six weeks of age. A1: Diagram of micro CT scanning of mandibular incisors. The perpendicular cutting plane, from root to tip along the three-dimensional incisor, was used to measure the enamel area, and two panels, representative of different sites, are shown in plane 1 and plane 2. Plane 1′ is a magnified view of plane 1. Enamel is coloured red, and dentin is coloured green. **A2** and **A3**: Micro CT scan of incisors from offspring of control and diabetic dams at three weeks (**A2**) and six weeks (**A3**) of age. Arrows indicate the enamel area. (**B**–**E**) Measurements of the cross-sectional area of enamel (**B**: three weeks; **D**: six weeks) and mineral density (**C**: three weeks; **E**: six weeks) are presented as means. *P < 0.05 vs. control.

The biological parameters of diabetic mothers and offspring are presented in Supplementary Figure S1. For tooth morphogenesis, the incisors of the offspring of diabetic dams at three weeks of age were smaller than those of control animals and had opaque spots (Fig. S1F). In addition, the expression of ameloblastin (AMBN) and amelogenin (AMGN) in ameloblasts was downregulated relative to that of control offspring (Fig. S1G). Micro computed tomography (CT) scanning (Fig. 1A) showed that the enamel of incisors was thinner, with less volume, in offspring of diabetic dams compared to that in controls at three weeks (Fig. 1A2 and B) and six weeks (Fig. 1A3 and D) of age. The mineral density of enamel also showed a modest reduction in the offspring of diabetic dams compared to that in control offspring (Fig. 1C and E). These data indicated that maternal diabetes affects enamel morphogenesis in offspring.

### High-glucose environments inhibit DESCs proliferation and self-renewal via modulation of APEX1 expression

**Figure 2:**
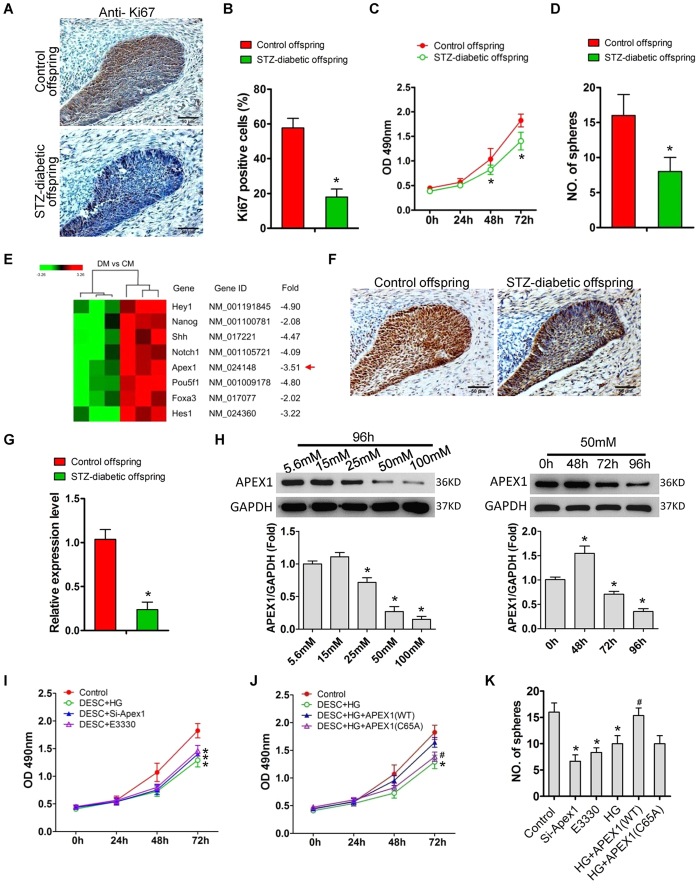
*Apex1* downregulation is involved in maternal diabetes-induced suppression of cell proliferation and self-renewal. (**A**,**B**) Ki67 immunostaining (**A**) and quantitative analysis of Ki67-positive cells (**B**) in the LaCL from neonates of control and diabetic dams. (**C**,**D**) Growth curve (**C**) and colony-forming ability (**D**) of dental epithelial stem cells (DESCs) isolated from neonates of control and diabetic dams. (**E**) Microarrays showed that *Apex1* was downregulated in the mandibular labial incisor cervical loops of offspring from diabetic dams compared to those of controls (DM: offspring of diabetic mothers; CM: offspring of control mothers). (**F**,**G**) Immunostaining (**F**) and real-time polymerase chain reaction indicated (**G**) significant downregulation of *Apex1* in the offspring of diabetic dams compared to those of controls. (**H**) *In vitro*, high glucose treatment of primary DESCs also significantly inhibited APEX1 expression in a dose- and time-dependent manner. (**I**–**K**) DESCs treated with high glucose, *Apex1* knock-down, or with E3330 to inhibit APEX1 redox function were significantly impaired for proliferation (**I**) and colony-forming ability (**K**). Overexpression of APEX1^WT^, but not APEX1^C65A^, attenuated the suppression of proliferation (**J**) and colony-forming ability (**K**) induced by high glucose treatment. *P < 0.05 vs. control; ^#^P < 0.05 vs. high glucose.

Previous studies have shown that DESCs present in the LaCL at the proximal end of the incisor give rise to highly proliferative, transit-amplifying (T-A) cells that differentiate into enamel-secreting ameloblasts^24^,^25^,^26^,^27^. Therefore, in this study, we focused on changes in the DESCs of offspring to determine the effects of maternal diabetes on offspring enamel development. Immunostaining showed that the number of Ki67-positive cells in the cervical loops of neonates with diabetic mothers was significantly lower than that of control offspring (Fig. 2A and B), and that primary DESCs, isolated from neonates with diabetic mothers, showed significantly slower growth (Fig. 1C) and a marked decrease in colony forming ability (Fig. 1D) compared to those of control offspring. To determine the molecular mechanism of offspring DESCs modulation in response to maternal diabetes, we performed genome-wide gene expression profiling of the LaCL, comparing control neonates and those from diabetic dams. Maternal diabetes resulted in the upregulation of 51 genes and the downregulation of 107 genes (Supplementary Table 2, Fig. S2). KEGG pathway mapping of differentially expressed genes showed that several signalling pathways, including the Notch and TGF-beta signalling pathways, were involved in aberrant tooth development induced by maternal diabetes (Fig. S2). Among the differentially expressed genes, *Apex1* was significantly downregulated in the LaCL of neonates with diabetic mothers, when compared to that of controls (Fig. 2E). Immunostaining (Fig. 2F) and real-time polymerase chain reaction (PCR) (Fig. 2G) confirmed the downregulation of *Apex1* in the offspring of diabetic dams. *In vitro*, primary DESCs were treated with high glucose to simulate hyperglycaemia induced by maternal diabetes *in vivo*. Results showed that high glucose downregulated APEX1 expression in DESCs in a dose- and time-dependent manner (Fig. 2H). The osmotic control of glucose and mannitol did not induce significant changes in APEX1 expression. In DESCs, high glucose, APEX1 knockdown, or E3330 inhibition of APEX1 redox function significantly suppressed DESCs proliferation and self-renewal (Fig. 2I and K). Mannitol did not induce any changes in DESCs proliferation or colony formation. Exogenous overexpression of wild-type APEX1 (APEX1^WT^), via plasmid transfection, attenuated the suppression of proliferation and sphere formation induced by high glucose (Fig. 2J and K). However, these effects were not observed in DESCs overexpressing the redox-deficient APEX1 (APEX1^C65A^) (Fig. 2J and K).

### In a high-glucose environment, APEX1 regulates *Oct4* and *Nanog* expression in DESCs via DNA methylation

**Figure 3:**
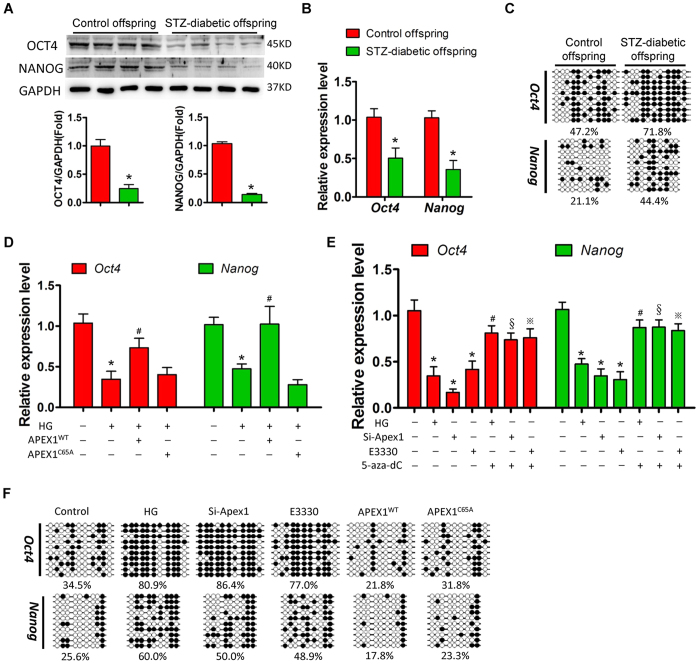
High glucose treatment inhibits *Oct4* and *Nanog* expression via APEX1-mediated DNA methylation. (**A**,**B**) Western blot (**A**) and real-time PCR (**B**) analyses of OCT4 and NANOG expression in the cervical loops of offspring of control and diabetic dams; *P < 0.05 vs. control; (**C**) Sodium bisulphite sequencing analyses of DNA methylation levels of the *Oct4* promoter (from −533 to −34) and *Nanog* promoter (from −672 to −332) in the cervical loops of offspring from control and diabetic dams. (**D**) High glucose treatment decreased the expression of *Oct4* and *Nanog* in dental epithelial stem cells (DESCs), and overexpression of APEX1^WT^, but not APEX1^C65A^, reversed the downregulation of *Oct4* and *Nanog* induced by high glucose treatment; *P < 0.05 vs. control; ^#^P < 0.05 vs. high glucose. (**E**) Real-time PCR showed decreased expression of *Oct4* and *Nanog* in response to high glucose treatment, *Apex1* knockdown, or inhibition of APEX1 redox function by E3330 in primary DESCs; treatment with the DNMT inhibitor 5-aza-dC significantly reversed the downregulation of *Oct4* and *Nanog*; *P < 0.05 vs. control; ^#^P < 0.05 vs. HG; ^§^P < 0.05 vs. Si-*Apex1*; ^※^P < 0.05 vs. E3330. (**F**) Sodium bisulphite sequencing showed an increase in *Oct4* and *Nanog* promoter DNA methylation levels in DESCs treated with high glucose, APEX1-specific siRNA or E3330. Overexpression of APEX1^WT^, but not APEX1^C65A^, decreased DNA methylation levels of the *Oct4* and *Nanog* promoters.

*Oct4* and *Nanog* are two key transcription factors that are known to regulate stem cell proliferation and self-renewal^28^. Here, we found that OCT4 and NANOG expression was significantly downregulated in the offspring of diabetic dams compared to that in control offspring (Fig. 3A and B). Sodium bisulphite sequencing showed higher DNA methylation levels in the *Oct4* and *Nanog* gene promoter regions in the offspring of diabetic dams compared to that in the control group (Fig. 3C), indicating that DNA methylation might be involved in the regulation of *Oct4* and *Nanog* expression in response to maternal diabetes.

In primary DESCs, *in vitro* high glucose treatment also resulted in significant downregulation of *Oct4* and *Nanog* (Fig. 3D), whereas mannitol treatment did not induce significant changes in *Oct4* and *Nanog* expression. Exogenous overexpression of wild-type APEX1 (APEX1^WT^), but not redox-deficient APEX1 (APEX1^C65A^), significantly attenuated the decrease in expression of *Oct4* and *Nanog* induced by high glucose (Fig. 3D). In contrast, *Apex1* knockdown or redox inhibition by E3330 resulted in significant downregulation of *Oct4* and *Nanog* (Fig. 3E), and this downregulation was reversed by 5-aza-dC treatment, which is an inhibitor of DNA methyltransferase (DNMT) (Fig. 3E). Sodium bisulphite sequencing showed that high glucose treatment or APEX1 inhibition enhanced DNA methylation of the *Oct4* and *Nanog* promoters (Fig. 3F). Furthermore, overexpression of wild-type APEX1, but not redox-deficient APEX1, significantly decreased the DNA methylation levels of these promoters (Fig. 3F).

### APEX1 is involved in DNMT1 upregulation and DNA hypermethylation induced by high glucose

**Figure 4:**
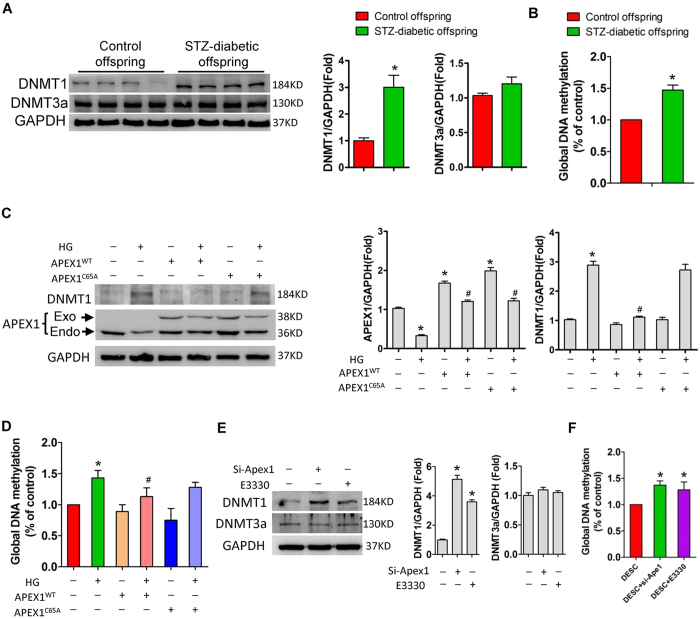
Impaired APEX1 contributes to DNMT1 upregulation and DNA hypermethylation induced by high glucose treatment in dental epithelial stem cells (DESCs). (**A**) Western blot analysis of DNMT1 and DNMT3a levels in the cervical loops of offspring from control and diabetic dams; *P < 0.05 vs. control. (**B**) Global DNA methylation levels in the cervical loop of offspring from control and diabetic dams; *P < 0.05 vs. control. (**C**) DESCs treated with high glucose showed significant upregulation of DNMT1 and overexpression of APEX1^WT^, whereas redox-deficient APEX1^C65A^ attenuated the DNMT1 upregulation induced by high-glucose treatment; *P < 0.05 vs. control; ^#^P < 0.05 vs. high glucose. (**D**) High glucose treatment induced a significant increase in global DNA methylation levels and overexpression of APEX1^WT^, whereas redox-deficient APEX1^C65A^ attenuated global DNA hypermethylation induced by high glucose treatment; *P < 0.05 vs. control; ^#^P < 0.05 vs. high glucose. (**E**) Western blotting showed that *Apex1* knockdown or inhibition of redox activity by the inhibitor E3330 enhanced DNMT1 expression, but did not affect DNMT3a expression; *P < 0.05 vs. control. (**F**) *Apex1* knockdown or inhibition of redox activity by the inhibitor E3330 significantly increased global DNA methylation levels; *P < 0.05 vs. control.

DNA methyltransferase is responsible for cytosine methylation in mammals and has a role in gene silencing^29^,^30^. Here, we found that *Dnmt1* and *Dnmt3a* were expressed in DESCs, whereas *Dnmt3b* was undetectable (Fig. S2). In the offspring of diabetic dams, there was a significant increase in the expression of DNMT1, but not DNMT3a, and in global DNA methylation levels in LaCL, relative to those of control offspring (Fig. 4A and B). *In vitro*, high glucose treatment significantly increased DNMT1 expression in DESCs (Fig. 4C); overexpression of wild-type APEX1 (APEX1^WT^), but not redox-deficient APEX1 (APEX1^C65A^), significantly attenuated the increase in DNMT1 expression induced by high-glucose treatment (Fig. 4C). In contrast, mannitol treatment did not induce significant changes in *Dnmt1* and *Dnmt3a* expression. In addition, global DNA methylation levels were increased in DESCs in response to high glucose treatment (Fig. 4D), and overexpression of APEX1^WT^, but not redox-deficient APEX1^C65A^, attenuated this increase in global DNA methylation (Fig. 4D). In contrast, knockdown of *Apex1* or inhibition of APEX1 redox activity by E3330 also significantly upregulated DNMT1 expression and increased global DNA methylation levels (Fig. 4E and F).

### Extracellular signal-regulated kinase (ERK) and Jun amino-terminal kinase (JNK) signal activation is involved in DNMT1 upregulation induced by high glucose or APEX1 inhibition

**Figure 5:**
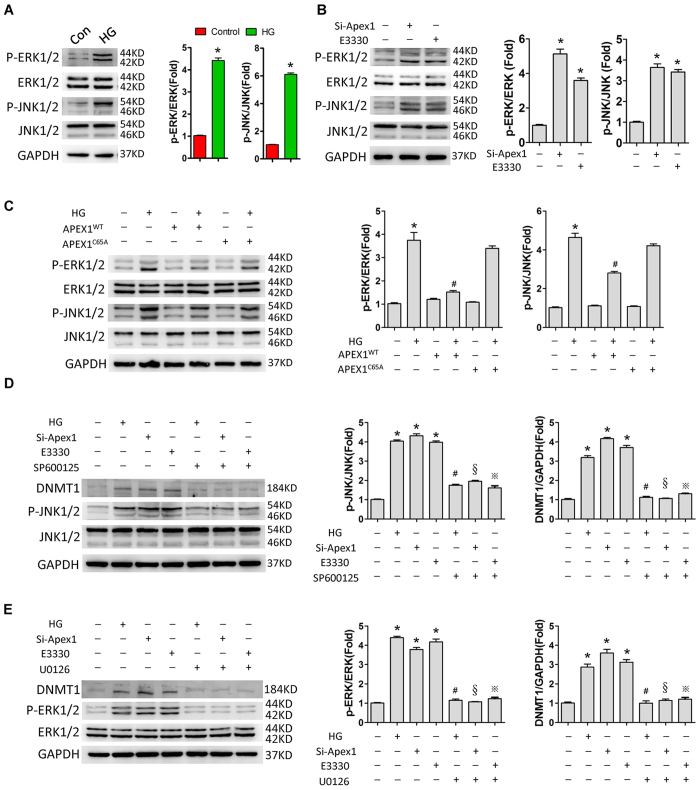
High glucose treatment or APEX1 inhibition induces DNMT1 expression by activating ERK and JNK signalling pathways. (**A**,**B**) High glucose treatment (**A**) and *Apex1* knockdown or inhibition of APEX1 redox function by E3330 (**B**) activated ERK and JNK signalling. (**C**) Overexpression of APEX1^WT^, but not redox-deficient APEX1^C65A^, attenuated the activation of ERK and JNK signalling induced by high glucose treatment. (**D**,**E**) The specific JNK inhibitor SP600125 (**D**) or the ERK inhibitor U0126 (**E**) reversed DNMT1 upregulation in response to high glucose treatment, *Apex1* knockdown, or inhibition of APEX1 redox function by E3330. *P < 0.05 vs. control; ^#^P < 0.05 vs. HG; ^§^P < 0.05 vs. Si-*Apex1*; ^※^P < 0.05 vs. E3330.

As described above, high glucose or APEX1 inhibition (gene knockdown or redox activity inhibition by E3330) upregulated DNMT1 expression, and overexpression of wild-type APEX1 attenuated the upregulation of DNMT1 induced by high glucose. Next, we found that high glucose treatment, *Apex1* knockdown, or APEX1 redox activity inhibition activated ERK and JNK signalling (Fig. 5A and B), and that overexpression of APEX1^WT^, but not redox-deficient APEX1^C65A^, attenuated this activation (Fig. 5C). Treatment of DESCs with the JNK inhibitor SP600125 or the ERK inhibitor U0126 reversed the upregulation of DNMT1 induced by high glucose or APEX1 inhibition (Fig. 5D and E).

## Discussion

A number of studies have shown that the maternal environment, in particular hyperglycaemia during pregnancy, can alter foetal development, affecting organ formation and increasing the risk of diseases such as neural tube defects, cardiovascular disease, obesity, diabetes, and cancer in the offspring via epigenetic mechanisms^31^,^32^,^33^,^34^,^35^,^36^,^37^,^38^,^39^. In the present study, we established a maternal gestational diabetes rat model to determine its effects on tooth development in offspring and to study the mechanisms associated with these effects. Our results indicated that exposure to a high glucose environment *in utero* inhibited DESCs proliferation and self-renewal via downregulation of *Apex1* expression and consequently DNA hypermethylation of the *Oct4* and *Nanog* promoters. Moreover, we found that *Apex1* downregulation resulted in upregulation of DNMT1 expression, a key enzyme responsible for DNA methylation, through activation of the ERK and JNK signalling pathways. Our study is the first to show that suppression of DESCs proliferation and self-renewal in offspring might result from *Apex1* downregulation induced by maternal diabetes, which might lead to activation of DNMT1 and hypermethylation of the *Oct4* and *Nanog* promoters. These results indicate that APEX1 acts as a critical regulator during tooth development and show how maternal diabetes can affect DESCs proliferation and self-renewal in the offspring through an epigenetic mechanism.

There is now substantial evidence from epidemiological studies and animal models indicating that diabetes mellitus affects tooth development including tooth eruption and enamel mineralization^7^,^8^,^9^,^10^. However, understanding of the cellular and molecular mechanisms though which *in utero* hyperglycaemia alters this process is lacking. Here, we found that maternal diabetes suppresses DESCs proliferation and self-renewal in the LaCL, the stem cell niche for incisors that allows them to grow continuously. DESCs located in the cervical loop give rise to four cell lineages: inner enamel epithelium (the ameloblast cell lineage), stratum intermedium, stellate reticulum, and outer enamel epithelium^40^,^41^. Previous studies using knockout mice have confirmed that inhibition of DESCs proliferation and self-renewal causes tooth defects^41^,^42^. In terms of pluripotency, *Oct4* and *Nanog* play important roles in the proliferation and self-renewal of stem cells^28^. Previous reports have shown that *Oct4* and *Nanog* are expressed in the dental epithelium and mesenchyme and regulate cell proliferation, stemness, and differentiation in stem cells^43^,^44^. Nakagawa *et al*., using tooth germ organ culture and *Oct4* siRNA, also found that OCT4 was critical for tooth epithelial stem cell proliferation and self-renewal^45^. In our study, *Oct4* and *Nanog* were downregulated by maternal diabetes, which might result in the suppression of DESCs proliferation and self-renewal.

DNA methylation is a key epigenetic mechanism that regulates gene expression and chromosomal stability. Hypermethylation of promoter CpG islands usually leads to the downregulation of gene expression. DNA methylation is accomplished by DNMT including DNMT1, DNMT3a, and DNMT3b. DNMT1 is the most abundant DNMT and is considered to be key for maintenance of methyltransferase activity in mammals. All of these DNMTs are essential for embryonic development and critical for the maintenance of stem cell properties^29^. Many factors can alter the expression or activity of DNMT, such as hyperglycaemia and oxidative stress, which in turn modulate DNA methylation and gene expression^46^,^47^. Thus, foetal exposure to hyperglycaemia during the critical stages of development can persistently alter the pattern of DNA methylation, resulting in abnormalities in organ systems such as bone and the nervous system^48^,^49^. Therefore, we hypothesized that maternal diabetes could affect tooth development in offspring via aberrant DNA methylation. In our model, sodium bisulphite sequencing confirmed that the *Oct4* and *Nanog* promoters were hypermethylated in the cervical loop of neonates from diabetic mothers, compared to those of control offspring, consistent with the downregulation of *Oct4* and *Nanog* expression in the offspring of diabetic dams. DNMT1 expression was also increased in these offspring. These results indicate that maternal diabetes affects tooth development in offspring through DNA methylation, which in turn modulates gene expression. To our knowledge, our data are the first to indicate that maternal diabetes modulates DNA methylation in the tooth tissue of offspring. Further investigation is necessary to understand the mechanism of aberrant DNA methylation that is induced by maternal diabetes, in the developing teeth of offspring.

APEX1 is a multifunctional protein involved in apurinic/apyrimidinic endonuclease DNA base excision repair activity and in modulating the redox status of transcription factors such as NF-kappa B, Egr-1, p53, AP-1, CREB, HIF-alpha, and members of the Pax family^19^,^20^. The two functional domains of APEX1 are distinct but overlapping: its DNA repair function is associated with the C-terminus, whereas its redox function is associated with the N-terminus. APEX1 is important for normal embryonic development; mouse embryos that do not express APEX1 die on embryonic day 6.5^21^. APEX1 was also found to play a role in the senescence of mesenchymal stem cells and in the hematopoietic differentiation of embryonic stem cells^50^,^51^. In addition, our previous study showed that APEX1 regulates the osteo/odontogenic differentiation of dental papilla cells through its redox function^23^. However, the role of APEX1 in dental epithelial stem cells is largely unknown. In the present study, *Apex1* knockdown or inhibition of redox activity suppressed proliferation and self-renewal in primary DESCs, and overexpression of *Apex1* significantly increased DESCs viability and colony-forming ability. This indicates that APEX1 plays a critical role during tooth development and that it might be a key target through which maternal diabetes affects offspring tooth development. Furthermore, we found that APEX1 regulates *Oct4* and *Nanog* expression through DNA methylation of their promoters. In addition, treatment with 5-aza-dC, a DNA methyltransferase inhibitor, reversed the downregulation of *Oct4* and *Nanog* induced by high glucose or APEX1 inhibition. These data suggest that APEX1 regulates the expression of *Oct4* and *Nanog* by modulating DNA methylation. As described above, DNMT1 is critical for DNA methylation, and our results show that DNMT1 was activated in the offspring of diabetic mothers. Previous reports have shown that APEX1 can inhibit *Dnmt1* expression or enzymatic activity via PARP1, resulting in DNA demethylation^52^. Therefore, we speculate that APEX1 might be a key regulator of DNA hypermethylation and the upregulation of DNMT1 expression induced by maternal diabetes.

Loss or gain of function analyses showed that overexpression of wild-type APEX1, but not redox-deficient APEX1, significantly decreased DNMT1 expression and methylation of the *Oct4* and *Nanog* promoters. In contrast, inhibition of redox function by E3330 or siRNA knockdown significantly increased DNMT1 expression and DNA methylation of the *Oct4* and *Nanog* promoters. These data indicate that the redox function of APEX1 might be responsible for the regulation of *Dnmt1* expression and DNA methylation. To date, several signalling pathways have been found to be involved in *Dnmt1* transcription, including the ERK and JNK signalling pathways^53^,^54^. In the present study, the ERK inhibitor U0126 or the JNK inhibitor SP600125 significantly reversed DNMT1 activation induced by *Apex1* knockdown or redox activity inhibition. These data suggest that APEX1 modulates *Dnmt1* expression and DNA methylation, mainly via its redox function and through activation/inactivation of ERK and JNK signalling pathways.

In summary, we demonstrated that maternal diabetes can result in the suppression of DESCs proliferation and self-renewal in offspring as a result of *Apex1* downregulation. This was shown to be mediated by increased DNMT1 expression and hypermethylation of *Oct4* and *Nanog* promoters. Our data suggest a new mechanism through which *Apex1* mediates the tooth development in maternal diabetes-affected offspring, and indicates a direct link between maternal diabetes and epigenetic silencing of genes such as *Oct4* and *Nanog*. The results of this study also suggest novel targets for strategies to prevent or treat tooth hypoplasia in addition to valuable information for tissue engineering to regenerate teeth.

## Methods

All experiments were conducted in accordance with a protocol approved by the Committee of Ethics of Sichuan University.

### Animal model

Diabetes was induced in pregnant Sprague-Dawley rats, identified by the presence of a copulation plug after mating (E0.5), on day 9.5 of gestation using a single intraperitoneal injection of streptozotocin (STZ (Sigma-Aldrich, St. Louis, USA); 75 mg/kg body weight in 0.1 mol/L citrate buffer [pH4.5]). STZ is a pancreatic beta-cell toxin that is widely used to experimentally manipulate insulin levels, and a rodent model of STZ-induced diabetes has been accepted internationally for use in diabetic embryopathy research^55^,^56^,^57^,^58^. Plasma glucose concentrations were measured on days 10.5 and 11.5 of pregnancy, and only those animals with plasma glucose levels greater than 15 mmol/L were included in this study. Therefore, the developing embryos were exposed to a hyperglycaemic environment from the initiation of tooth development at approximately embryonic day 10.5^59^. The diabetic status of rats was confirmed every two days until delivery. The neonates were euthanized, and lower LaCL tissues were collected. LaCL tissues from the same litter were pooled for DNA, RNA, and protein isolation. A litter was considered an experimental unit. All animals were allowed free access to food and water and maintained in a temperature and light controlled room at 21 °C with a 12-h light cycle. In this study, male offspring were selected from each litter for use in subsequent experiments.

### Genomic DNA and total RNA isolation

Genomic DNA and total RNA were isolated from pooled LaCL tissues or primary DESCs using a DNeasy Blood & Tissue Kit (Qiagen, Hilden, Germany) and RNAiso Reagent (TaKaRa Biotechnology, Shiga, Japan) according to the manufacturers’ instructions.

### Microarray gene expression

Whole Rat Genome Oligonucleotide 4 × 44 k Microarrays (Agilent, CA, USA) were used to measure gene expression. A p value of less than 0.05 and a mean expression change of greater than 2-fold was considered statistically significant and these genes were used for further analysis. Gene ontology (GO) and signalling pathway analyses of differentially expressed genes were analysed using the DAVID Functional Annotation Tool^60^. Three experimental units of offspring from control and diabetic dams were used for microarray analysis (n = 3).

### Primary DESCs culture and treatment

Primary DESCs culture was performed as previously described^61^. At least three independent experiments were performed, with at least three pooled litters in each group. For high glucose treatment, primary DESCs were maintained in medium containing concentrations of d-glucose ranging from 5.6 mM (normal glucose) to 100 mM, and the same concentrations of mannitol were used as equiosmolar controls. Unless specified in the main text, medium containing 50 mM d-glucose was used for the high glucose treatment. Four days after high glucose treatment, the cells were treated with an inhibitor for three days. For inhibitor treatment, cells were exposed to 5-aza-2′-deoxycytidine (5-aza-dC, a DNA demethylation reagent (Selleck Chemicals, China)), E3330 (an inhibitor of APEX1 redox activity (Sigma-Aldrich, USA)), U0126 (an ERK/MAPK inhibitor (Selleck Chemicals, China)), or SP600125 (a JNK/MAPK inhibitor (Selleck Chemicals, China)), all at concentrations of 5 μM. All inhibitors were dissolved in dimethyl sulphoxide (DMSO, Sigma-Aldrich, USA). Control cells received an equal amount of DMSO. In total, cells were exposed for one week to high glucose medium and for three days to inhibitors. All treatments were refreshed every 2 days. All experiments were performed in at least triplicate.

### Cell growth and colony formation assays

Cell growth was determined using a CCK-8 Assay (Dojindo Molecular Technologies, Kumamoto, Japan). To determine colony formation, DESCs were dissociated into single cells and seeded into a 24-well plate at a density of 200 cells per well. After two weeks, colonies were Giemsa stained and counted. All experiments were performed in triplicate and repeated at least three times.

### *Apex1* knockdown and overexpression

Knockdown of *Apex1* (NCBI Reference Sequence: NM_024148.1) was accomplished by RNA interference. Oligo sequences containing the RNA interference target were synthesized, annealed, and ligated into the pLKD-CMV-G&PR-U6-shRNA lentiviral vector (Neuronbiotech, Shanghai, China). The most efficient shRNA sequences for knockdown were the rat *Apex1* shRNA pair: 5′- CCGGGGTGATTGTGGCTGAATTTGACTCGAGTCAAATTCAGCCACAATCACCTTTTTTG-3′ (sense) and 5′- AATTCAAAAAAGGTGATTGTGGCTGAATTTGACTCGAGTCAAATTCAGCCACAATCACC-3′ (antisense). The scrambled shRNA pair was as follows: 5′-GATCCCCTTCTCCGAACGTGTCACGTTTCAAGAGAACGTGACACGTTCGGAGAATTTTTGGAAA-3′ (sense), and 5′-AGCTTTTCCAAAAATTCTCCGAACGTGTCACGTTCTCTTGAAACGTGACACGTTCGGAGAAGGG-3′ (antisense). The underlined sequences indicate hairpins.

To overexpress APEX1, the entire APEX1 coding sequence (wild-type APEX1, APEX1^WT^) and redox activity mutant (APEX1^C65A^, with APEX1 redox activity attributed to the Cys65 residue^20^,^62^) were verified by DNA sequencing and cloned into the pLOV-EF1a-PuroR-CMV-3FLAG plasmid. The plasmids were packaged, and lentiviruses were used to transduce DESCs.

### Immunostaining

Offspring were collected at E15.5, E17.5, and P0.5. Paraffin sections were prepared and immunostaining was performed as previously described^23^. Primary antibodies against Ki67 (ab15580, Abcam), AMBN (sc-50534, Santa Cruz), AMGN (sc-32892, Santa Cruz), and APEX1 (ab194, Abcam) were used. To determine the cell proliferation index, Ki67-positive cells in the cervical loop of each tissue section were counted. At least five serial sections from each animal were examined, and three animals from different litters (n = 3) were included in each group.

### Real-time PCR and western blotting

Real-time RT-PCR and western blotting were conducted as previously described^21^. PCR primer sequences are shown in Supplementary Table S1. Primary antibodies against APEX1 (ab194, Abcam), DNMT1 (sc-10222, Santa Cruz), ERK (#4695, Cell Signaling Technology), P-ERK (#4370, Cell Signaling Technology), JNK (#9258, Cell Signaling Technology), P-JNK (#4668, Cell Signaling Technology), and GAPDH (200306-7E4, ZEN) were used. Images were captured with an Image Quant LAS 4000 Mini (GE Healthcare Life Sciences), and proteins on blots were quantified by scanning densitometry (ImageQuant TL, GE Healthcare Life Sciences). Experiments were performed independently for each sample, and at least three technical replicates were performed for each of the treated samples and controls.

### Sodium bisulphite sequencing

Genomic DNA was treated with bisulphite and purified for PCR as previously described^63^. The primers for sequencing the *Oct4* and *Nanog* promoters were as follows: *Oct4*, 5′-AGGTTTTTTTGAATTTGAAGTTAG-3′ and 5′-CAAAACTAAACAACCACTCCAC-3′ (bp −533 to −34, 11 CG), and *Nanog*, 5′-GAGTTGTTGGTTTTTAGATAGGTTG-3′ and 5′-ACACTTATAAACAAAAATAATTTTCCTC-3′ (bp −672 to −332, 9 CG). The PCR products were gel extracted and ligated into a pGEM-T vector using the TA cloning system. Ten separate clones were selected for sequencing analysis.

### Global DNA methylation analysis

Genomic DNA methylation was quantified using the Methylamp Global DNA Methylation Quantification Ultra Kit (Epigentek, Brooklyn, USA) according to the manufacturer’s instructions. The amount of DNA methylation (percent methylation) was calculated using the following formula: percent methylation = [OD (sample − negative control) × GC content]/[OD (positive control − negative control) × 10] × 100. The GC content for rat genomic DNA was 42%. Experiments were repeated at least three times.

### Micro CT

At three or six weeks, the male offspring of diabetic and control mothers were sacrificed using intraperitoneally injected ketamine and xelazine, and mandibles were collected for micro CT analysis (n = 3 pups/group; and, in each group, a total of three separate pups from the same litter were selected randomly). Micro CT scanning of mandibles was performed using a high-resolution scanner (Y. Cheetah, YXLON International GmbH, Germany). Measurements of the incisor enamel area and mineral density were obtained as previously described^9^.

### Statistical analysis

All data are presented as the mean value ± standard deviation for each group. Statistical significance of differences between experimental groups was determined initially by a *t* test or analysis of variance (ANOVA), followed by a Bonferroni test when needed. P < 0.05 was considered statistically significant.

## Additional Information

**How to cite this article**: Chen, G. *et al*. Maternal diabetes modulates dental epithelial stem cells proliferation and self-renewal in offspring through apurinic/apyrimidinicendonuclease 1-mediated DNA methylation. *Sci. Rep.* **7**, 40762; doi: 10.1038/srep40762 (2017).

**Publisher's note:** Springer Nature remains neutral with regard to jurisdictional claims in published maps and institutional affiliations.

## Supplementary Material

Supplementary File

## References

[b1] Galtier F. Definition, epidemiology, risk factors. Diabetes Metab 36, 628–651 (2010). 21163426 10.1016/j.diabet.2010.11.014

[b2] Wren C., Birrell G. & Hawthorne G. Cardiovascular malformations in infants of diabetic mothers. Heart 89, 1217–1220 (2003). 12975424 10.1136/heart.89.10.1217 PMC1767924

[b3] Dheen S. T. . Recent studies on neural tube defects in embryos of diabetic pregnancy: an overview. Curr Med Chem 16, 2345–2354 (2009). 19519395 10.2174/092986709788453069

[b4] Hokke S. N. . Altered ureteric branching morphogenesis and nephron endowment in offspring of diabetic and insulin-treated pregnancy. PLoS One 8, e58243 (2013). 23516451 10.1371/journal.pone.0058243 PMC3596403

[b5] Clausen T. D. . High prevalence of type 2 diabetes and pre-diabetes in adult offspring of women with gestational diabetes mellitus or type 1 diabetes: the role of intrauterine hyperglycemia. Diabetes Care 31, 340–346 (2008). 18000174 10.2337/dc07-1596

[b6] Catalano P. M. . The hyperglycemia and adverse pregnancy outcome study: associations of GDM and obesity with pregnancy outcomes. Diabetes Care 35, 780–786 (2012). 22357187 10.2337/dc11-1790 PMC3308300

[b7] Lal S. . Accelerated tooth eruption in children with diabetes mellitus. Pediatrics 121, e1139–1143 (2008). 18450858 10.1542/peds.2007-1486

[b8] Villarino M. E. . Alterations of tooth eruption and growth in pups suckling from diabetic dams. Pediatr Res. 58, 695–699 (2005). 16189195 10.1203/01.PDR.0000180599.54807.24

[b9] Yeh C. K. . Hyperglycemia and xerostomia are key determinants of tooth decay in type 1 diabetic mice. Lab Invest 92, 868–882 (2012). 22449801 10.1038/labinvest.2012.60 PMC4513945

[b10] Silva-Sousa Y. T., Peres L. C. & Foss M. C. Enamel hypoplasia in a litter of rats with alloxan-induced diabetes mellitus. Braz Dent J. 14, 87–93 (2003). 12964650 10.1590/s0103-64402003000200003

[b11] Alfaradhi M. Z. & Ozanne S. E. Developmental programming in response to maternal overnutrition. Front Genet 2, 27 (2011). 22303323 10.3389/fgene.2011.00027 PMC3268582

[b12] Galjaard S., Devlieger R. & Van Assche F. A. Fetal growth and developmental programming. J Perinat Med. 41, 101–105 (2013). 23314514 10.1515/jpm-2012-0020

[b13] Hanson M. . Developmental plasticity and developmental origins of non-communicable disease: theoretical considerations and epigenetic mechanisms. Prog Biophys Mol Biol. 106, 272–280 (2011). 21219925 10.1016/j.pbiomolbio.2010.12.008

[b14] Messerschmidt D. M., Knowles B. B. & Solter D. DNA methylation dynamics during epigenetic reprogramming in the germline and preimplantation embryos. Genes Dev 28, 812–828 (2014). 24736841 10.1101/gad.234294.113 PMC4003274

[b15] Marcho C. 1., Cui W. & Mager J. Epigenetic dynamics during preimplantation development. Reproduction 150, R109–120 (2015). 26031750 10.1530/REP-15-0180 PMC4529766

[b16] Feinberg A. P. . Personalized epigenomic signatures that are stable over time and covary with body mass index. Sci Transl Med 2, 49ra67 (2010). 10.1126/scitranslmed.3001262 PMC3137242 20844285

[b17] Flanagan J. M. . Temporal stability and determinants of white blood cell DNA methylation in the breakthrough generations study. Cancer Epidemiol Biomarkers Prev 24, 221–229 (2015). 25371448 10.1158/1055-9965.EPI-14-0767

[b18] Clarke-Harris R. . PGC1α promoter methylation in blood at 5–7 years predicts adiposity from 9 to 14 years (EarlyBird 50). Diabetes 63, 2528–2537 (2014). 24622795 10.2337/db13-0671

[b19] Tell G. . The many functions of APE1/Ref-1: not only a DNA repair enzyme. Antioxid Redox Signal11 601–620 (2009). 10.1089/ars.2008.2194 PMC2811080 18976116

[b20] Bhakat K. K., Mantha A. K. & Mitra S. Transcriptional regulatory functions of mammalian AP-endonuclease (APE1/Ref-1), an essential multifunctional protein. Antioxid Redox Signal 11, 621–638 (2009). 18715144 10.1089/ars.2008.2198 PMC2933571

[b21] Xanthoudakis S. . The redox/DNA repair protein, Ref-1, is essential for early embryonic development in mice. Proc Natl Acad Sci USA 93, 8919–8923 (1996). 8799128 10.1073/pnas.93.17.8919 PMC38569

[b22] Wang K. . Redox homeostasis: the linchpin in stem cell self-renewal and differentiation. Cell Death Dis 4, e537 (2013) 23492768 10.1038/cddis.2013.50 PMC3613828

[b23] Chen T. . Inhibition of Ape1 Redox Activity Promotes Odonto/osteogenic Differentiation of Dental Papilla Cells. Sci Rep 5, 17483 (2015). 26639148 10.1038/srep17483 PMC4671010

[b24] Mao J. J. & Prockop D. J. Stem cells in the face: tooth regeneration and beyond. Cell Stem Cell 11, 291–301 (2012). 22958928 10.1016/j.stem.2012.08.010 PMC4093804

[b25] Seidel K. . Hedgehog signaling regulates the generation of ameloblast progenitors in the continuously growing mouse incisor. Development 137, 3753–3761 (2010). 20978073 10.1242/dev.056358 PMC3049275

[b26] Juuri E. . Sox2+ stem cells contribute to all epithelial lineages of the tooth via Sfrp5+ progenitors. Dev Cell 23, 317–328 (2012). 22819339 10.1016/j.devcel.2012.05.012 PMC3690347

[b27] Biehs B. . BMI1 represses Ink4a/Arf and Hox genes to regulate stem cells in the rodent incisor. Nat Cell Biol 15, 846–852 (2013). 23728424 10.1038/ncb2766 PMC3735916

[b28] Loh Y. H. . The Oct4 and Nanog transcription network regulates pluripotency in mouse embryonic stem cells. Nat Genet 38, 431–440 (2006). 16518401 10.1038/ng1760

[b29] Smith Z. D. & Meissner A. DNA methylation: roles in mammalian development. Nat Rev Genet 14, 204–220 (2013). 23400093 10.1038/nrg3354

[b30] Siegfried Z. . DNA methylation represses transcription *in vivo*. Nat Genet 22, 203–206 (1999). 10369268 10.1038/9727

[b31] Yogev Y. & Visser G. H. Obesity, gestational diabetes and pregnancy outcome. Semin Fetal Neonatal Med 14, 77–84 (2009). 18926784 10.1016/j.siny.2008.09.002

[b32] Eriksson U. J., Cederberg J. & Wentzel P. Congenital malformations in offspring of diabetic mothers–animal and human studies. Rev Endocr Metab Disord 4, 79–93 (2003) 12618562 10.1023/a:1021879504372

[b33] JAMES L. MILLS. Malformations in Infants of Diabetic Mothers. Birth Defects Res A Clin Mol Teratol 88, 769–778 (2010) 20973049 10.1002/bdra.20757 PMC4158942

[b34] Correa A. . Diabetes mellitus and birth defects. Am J Obstet Gynecol 199, 237 e1-9 (2008) 18674752 10.1016/j.ajog.2008.06.028 PMC4916956

[b35] Wren C., Birrell G. & Hawthorne G. Cardiovascular malformations in infants of diabetic mothers. Heart 89, 1217–1220 (2003). 12975424 10.1136/heart.89.10.1217 PMC1767924

[b36] Wei D. & Loeken M. R. Increased DNA Methyltransferase 3b (Dnmt3b) -mediated CpG Island Methylation Stimulated by Oxidative StressInhibits Expression of a Gene Required for Neural Tube and Neural Crest Development in Diabetic Pregnancy. Diabetes 63, 3512–3522 (2014). 24834974 10.2337/db14-0231 PMC4171658

[b37] Vrachnis N. . Impact of maternal diabetes on epigenetic modifications leading to diseases in the offspring. Exp Diabetes Res. 2012, 538474 (2012). 23227034 10.1155/2012/538474 PMC3512252

[b38] Li C. C. . Maternal obesity and diabetes induces latent metabolic defects and widespread epigenetic changes in isogenic mice. Epigenetics 8, 602–611 (2013). 23764993 10.4161/epi.24656 PMC3857340

[b39] Quilter C. R. . Impact on offspring methylation patterns of maternal gestational diabetes mellitus and intrauterine growth restraint suggest common genes and pathways linked to subsequent type 2 diabetes risk. FASEB J. 28, 4868–4879 (2014). 25145626 10.1096/fj.14-255240

[b40] Harada H. . Localization of putative stem cells in dental epithelium and their association with Notch and FGF signaling. J Cell Biol. 147, 105–120 (1999). 10508859 10.1083/jcb.147.1.105 PMC2164976

[b41] Lin Y. . FGFR2 in the dental epithelium is essential for development and maintenance of the maxillary cervical loop, a stem cell niche in mouse incisors. Dev Dyn 238, 324–330 (2009). 18985768 10.1002/dvdy.21778 PMC3052783

[b42] Lapthanasupkul P. . Ring1a/b polycomb proteins regulate the mesenchymal stem cell niche in continuously growing incisors. Dev Biol 367, 140–153 (2012). 22562112 10.1016/j.ydbio.2012.04.029

[b43] da Cunha J. M. . Pluripotent stem cell transcription factors during human odontogenesis. Cell Tissue Res 353, 435–441 (2013). 23736381 10.1007/s00441-013-1658-y

[b44] Nakagawa E. . The novel expression of Oct3/4 and Bmi1 in the root development of mouse molars. Cell Tissue Res 347, 479–484 (2012). 22287043 10.1007/s00441-011-1310-7

[b45] Nakagawa E. . The novel function of Oct3/4 in mouse tooth development. Histochem Cell Biol 137, 367–376 (2012). 22159899 10.1007/s00418-011-0895-y

[b46] Soberanes S. . Particulate matter Air Pollution induces hypermethylation of the p16 promoter Via a mitochondrial ROS-JNK-DNMT1 pathway. Sci Rep 2, 275 (2012). 22355787 10.1038/srep00275 PMC3281276

[b47] Yang B. T. . Increased DNA methylation and decreased expression of PDX-1 in pancreatic islets from patients with type 2 diabetes. Mol Endocrinol 26, 1203–1212 (2012). 22570331 10.1210/me.2012-1004 PMC5416998

[b48] Salbaum J. M. & Kappen C. Diabetic embryopathy: a role for the epigenome? Birth Defects Res A Clin Mol Teratol 91, 770–780 (2011). 21538816 10.1002/bdra.20807 PMC3152694

[b49] Chen J. R. . Inhibition of fetal bone development through epigenetic down-regulation of HoxA10 in obese rats fed high-fat diet. FASEB J 26, 1131–1141 (2012). 22131269 10.1096/fj.11-197822

[b50] Heo J. Y. . Downregulation of APE1/Ref-1 is involved in the senescence of mesenchymal stem cells. Stem Cells 27, 1455–1462 (2009). 19492297 10.1002/stem.54

[b51] Zou G. M. . Ape1 regulates hematopoietic differentiation of embryonic stem cells through its redox functional domain. Blood 109, 1917–1922 (2007). 17053053 10.1182/blood-2006-08-044172

[b52] Gavin D. P., Chase K. A. & Sharma R. P. Active DNA demethylation in post-mitotic neurons: a reason for optimism. Neuropharmacology 75, 233–245 (2013). 23958448 10.1016/j.neuropharm.2013.07.036 PMC3864977

[b53] Chen Y. . Decreased ERK and JNK signaling contribute to gene overexpression in “senescent” CD4+CD28- T cells through epigenetic mechanisms. J Leukoc Biol. 87, 137–145 (2010). 19843577 10.1189/jlb.0809562 PMC2801623

[b54] Sunahori K. . The catalytic subunit of protein phosphatase 2A (PP2Ac) promotes DNA hypomethylation by suppressing the phosphorylated mitogen-activated protein kinase/extracellular signal-regulated kinase (ERK) kinase (MEK)/phosphorylated ERK/DNMT1 protein pathway in T-cells from controls and systemic lupus erythematosus patients. J Biol Chem 288, 21936–21944 (2013). 23775084 10.1074/jbc.M113.467266 PMC3724648

[b55] Eriksson U. J., Dahlström E. & Hellerström C. Diabetes in pregnancy. Skeletal malformations in the offspring of diabetic rats after intermittent withdrawal of insulin in early gestation. Diabetes 32, 1141–1145 (1983). 6360760 10.2337/diab.32.12.1141

[b56] Li X., Xu C. & Yang P. c-Jun NH2-terminal kinase 1/2 and endoplasmic reticulum stress as interdependent and reciprocal causation in diabetic embryopathy. Diabetes 62, 599–608 (2013). 22961085 10.2337/db12-0026 PMC3554374

[b57] Li X. . Oxidative stress-induced JNK1/2 activation triggers proapoptotic signaling and apoptosis that leads to diabetic embryopathy. Diabetes 61, 2084–2092 (2012). 22688338 10.2337/db11-1624 PMC3402327

[b58] Jawerbaum A. & White V. Animal models in diabetes and pregnancy. Endocr Rev 31, 680–701 (2010). 20534704 10.1210/er.2009-0038

[b59] Tucker A. & Sharpe P. The cutting-edge of mammalian development; how the embryo makes teeth. Nat Rev Genet 5, 499–508 (2004). 15211352 10.1038/nrg1380

[b60] Huang da W., Sherman B. T. & Lempicki R. A. Systematic and integrative analysis of large gene lists using DAVID bioinformatics resources. Nat Protoc 4, 44–57 (2009). 19131956 10.1038/nprot.2008.211

[b61] Chavez M. G. . Characterization of dental epithelial stem cells from the mouse incisor with two-dimensional and three-dimensional platforms. Tissue Eng Part C Methods 19, 15–24 (2013). 22742471 10.1089/ten.tec.2012.0232 PMC3522131

[b62] Zhu J. . Genistein induces apoptosis by stabilizing intracellular p53 protein through an APE1-mediated pathway. Free Radic Biol Med 86, 209–218 (2015). 26032169 10.1016/j.freeradbiomed.2015.05.030

[b63] Li Y. & Tollefsbol T. O. DNA methylation detection: bisulfite genomic sequencing analysis. Methods Mol Biol 791, 11–21 (2011). 21913068 10.1007/978-1-61779-316-5_2 PMC3233226

