# Supplementary material for: Maternal diabetes modulates dental epithelial stem cells proliferation and self-renewal in offspring through apurinic/apyrimidinicendonuclease 1-mediated DNA methylation
Source: Sci Rep. 2017 Jan 17;7:40762. doi: 10.1038/srep40762 (PMC5240105; doi:10.1038/srep40762)
Supplement: Supplementary File [file srep40762-s1.pdf]

Maternal diabetes modulates dental epithelial stem cells proliferation and self-renewal in offspring through apurinic/apyrimidinicendonuclease 1-mediated DNA methylation

Guoqing Chen[1,2], Jie Chen[1,2,3], Zhiling Yan[1,2,3], Ziyue Li[1,2], Mei Yu[1,2], Weihua Guo[1,2,4*], Weidong Tian[1,2,3*]

[1] State Key Laboratory of Oral Diseases, West China College of Stomatology, Sichuan University, Chengdu, 610041, P.R. China

[2] National Engineering Laboratory for Oral Regenerative Medicine, West China College of Stomatology, Sichuan University, Chengdu, 610041, P.R. China

[3] Department of Oral and Maxillofacial Surgery, West China College of Stomatology, Sichuan University, Chengdu, 610041, P.R. China

[4] Department of Pedodontics, West China College of Stomatology, Sichuan University, No.14, 3rd Section, Renmin South Road, Chengdu 610041, P.R. China

***Corresponding author:**

Weihua Guo, Department of Pedodontics, West China College of Stomatology, Sichuan University, No. 14, 3rd Section, Renmin South Road, Chengdu 610041, PR China. Tel/fax: +86 28 8550 3499. E-mail address: guoweihua943019@163.com

Wei-dong Tian, Department of Oral and Maxillofacial Surgery, West China College of Stomatology, Sichuan University, No.14, 3rd Section, Renmin South Road Chengdu 610041, PR China, Tel/fax: +86 28 8550 3499

E-mail address: drtwd@sina.com

Supplementary Table S1 Primer sequences for real-time PCR

Gene	Primer sequences	Product size
*Oct*4	5'-tgttcctgtcactgctctgg-3'<br>5'-cccctgtttgtgctttcaat-3'	164bp
Nanog	5'-cccaagctaaagctgtctgg-3',<br>5'-atctgctggaggctgaggta-3'	167bp
Dnmt1	5'-ccaccaccaagctggtctat-3',<br>5'-tacggccaagttaggacacc-3'	233bp
Apex1	5'-gggcagcaaagaaaactgag-3',<br>5'-cgggagtttgttctctgagc-3'	233bp
Dnmt3a	5'-ggggacaagaatgctaccaa-3',<br>5'-gagcttctcgacacacacca-3'	155bp
Dnmt3b	5'-gtgaagcggatgatggagat-3',<br>5'-cctccgagacttggtagctg-3',	230bp
beta-actin	5'-acggtcaggtcatcactatcg-3',<br>5'-ggcatagaggtctttacggatg-3'	225bp

Supplementary Table S2 List of differentially expressed genes in labial cervical loop regulated by maternal diabetes

Gene symbol	Fold change	Gene symbol	Fold change	Gene symbol	Fold change
Igfbp3	-12.33	*Arhgap15*	-2.36	*Tsfm*	2.01
Tgfbr2	-10.30	*Mmp3*	-2.36	*Slc25a47*	2.02
Igf2	-6.79	*Esr1*	-2.35	*Slco1c1*	2.06
Hey2	-5.82	*Gcgr*	-2.34	*Hnf1a*	2.08
Psen2	-5.66	*Idi2l*	-2.34	*Cnga1*	2.08
Msx1	-5.61	*LOC688302*	-2.32	*Olr491*	2.08
Psen1	-5.08	*Cd163*	-2.32	*Samd7*	2.10
Tgfb1i1	-5.04	*Wfikkn2*	-2.31	*RGD1562550*	2.11
Mmp20	-4.97	*Igf1r*	-2.30	*LOC680029*	2.13
Pparg	-4.96	*Rab3b*	-2.29	*Vom2r46*	2.13
Hey1	-4.90	*Kcnj1*	-2.29	*Olr32*	2.16
Pou5f1	-4.80	*LOC363337*	-2.28	*Il13ra2*	2.17
Shh	-4.47	*Sptlc3*	-2.28	*Hist1h3f*	2.19
Tmem213	-4.47	*Nov*	-2.27	*Btnl5*	2.23
Jag2	-4.47	*Il7r*	-2.24	*Olr670*	2.24
Notch3	-4.32	*Aqp9*	-2.21	*Olr140*	2.24
Notch4	-4.22	*Msx3*	-2.21	*RGD1561551*	2.24
Notch1	-4.09	*Ikzf3*	-2.20	*Ccng1*	2.25
Igfbp5	-3.84	*Rt1.aa*	-2.20	*Cplx1*	2.26
Tmco5b	-3.83	*Adam10*	-2.19	*LOC683581*	2.27
Igf2bp2	-3.78	*Igf1*	-2.19	*RT1-N1*	2.30
Bmp2	-3.73	*Zfp167*	-2.19	*Syt1*	2.31
Tet2	-3.72	*Corin*	-2.19	*Cyp2c23*	2.36
LOC690626	-3.60	*Scara5*	-2.18	*Cdh8*	2.36
LOC296778	-3.55	*RGD1310572*	-2.17	*Cnga2*	2.40
Bmp4	-3.52	*LOC686041*	-2.16	*Acsbg2*	2.46
Apex1	-3.51	*Cd74*	-2.15	*Rnd1*	2.52
Sst	-3.43	*Olr931*	-2.14	*Serpind1*	2.52
RT1-Db1	-3.40	*Hoxd9*	-2.14	*Acta1*	2.60
Inmt	-3.36	*Cma1*	-2.14	*Eaf2*	2.67
C7	-3.33	*Tbr1*	-2.13	*Myh8*	2.69
Olr804	-3.31	*Fcn1*	-2.13	*Dhrs7*	2.75
Hes1	-3.22	*Scrt1*	-2.13	*Myl1*	2.78
Jag1	-3.21	*Slc22a12*	-2.12	*LOC691272*	2.78
LOC690977	-3.05	*Cacng4*	-2.12	*LOC683156*	2.79
RT1-Ba	-2.96	*RGD1563185*	-2.11	*Nppb*	2.82
Ear11	-2.86	*Snhg11*	-2.10	*LOC100364769*	2.86
RGD1564324	-2.79	*Fgf10*	-2.08	*Omg*	2.89
Olr610	-2.73	*Psapl1*	-2.08	*Il24*	2.97

RGD1565166	-2.73	*Nanog*	-2.08	*Amy2*	3.12
Ctsm	-2.65	*LOC501296*	-2.07	*Klrk1*	3.14
Lect1	-2.56	*Pdia2*	-2.05	*Grm5*	3.15
Bmp3	-2.52	*Ceacam10*	-2.05	*Bank1*	3.28
Bcl11b	-2.52	*Dusp15*	-2.05	*RGD1559615*	3.31
Scn7a	-2.47	*Klra22*	-2.03	*Il1rapl2*	3.64
St18	-2.46	*Ly6g6e*	-2.03	*Amelx*	4.17
LOC245710	-2.45	*LOC689749*	-2.03	*Dnmt1*	4.47
Fam180a	-2.45	*Rnf17*	-2.03	*Fam19a4*	4.93
Tgfb2	-2.44	*Foxa3*	-2.02	*RT1-CE10*	5.21
Ibsp	-2.44	*Col6a1*	-2.02	*LOC287167*	5.46
RT1-Da	-2.43	*RT1-CE16*	-2.01	*Rhox9*	31.60
Hmgcs2	-2.43	*Masp1*	-2.01		
LOC690821	-2.40	*Ankrd33b*	-2.01		
Krt23	-2.39				

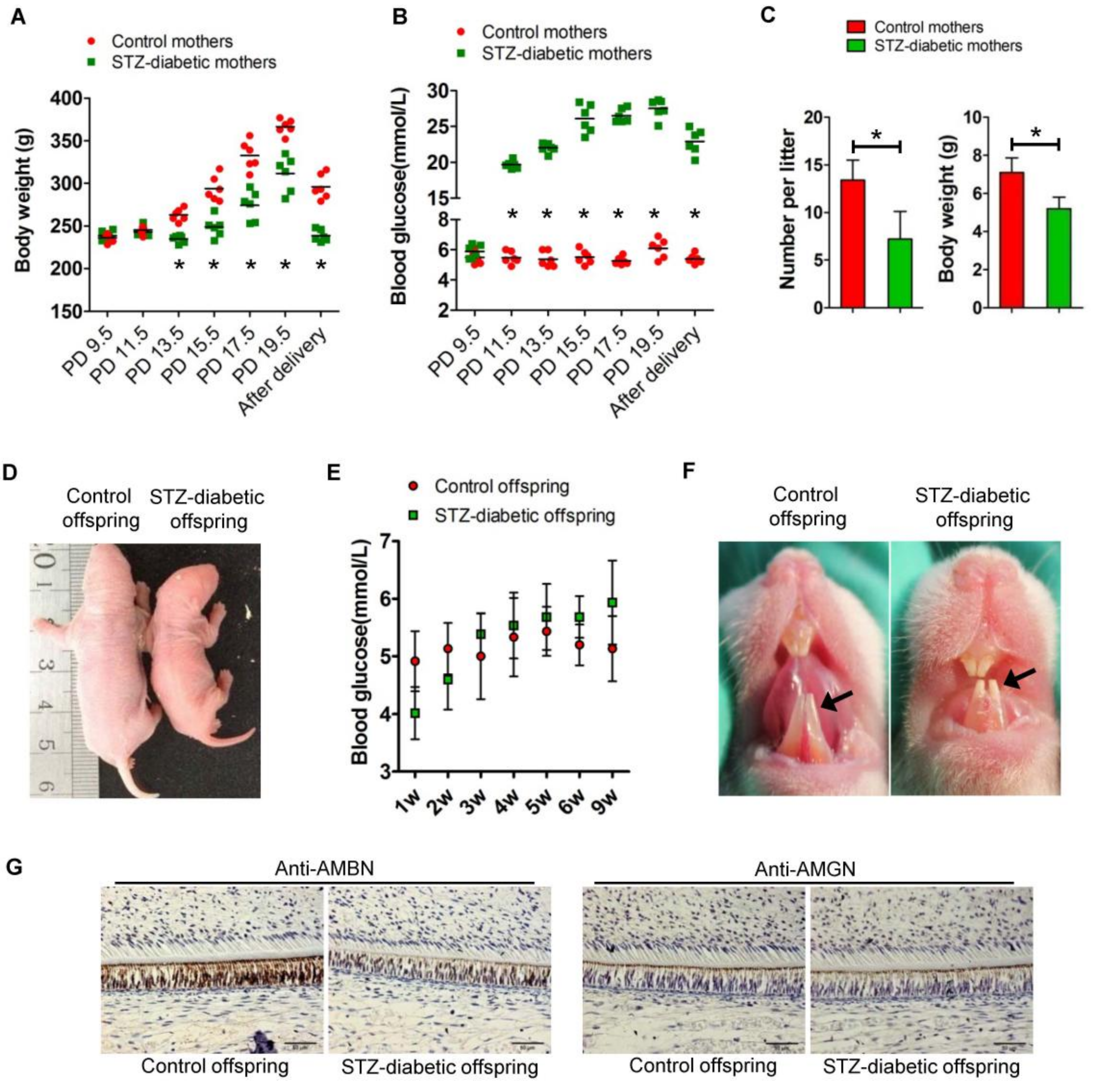

Supplementary Fig. S1 Physical parameters in diabetic mothers and offspring.

A and B: Body weights (A) and blood glucose levels (B) of control and diabetic mothers (PD: pregnant day), *P < 0.01 vs. control. C: Litter sizes and body weights of neonates of control and diabetic mothers; *P < 0.01 vs. control. D: Image of representative neonates from control and diabetic mothers. E: Blood glucose levels of offspring of control and diabetic dams from birth to nine weeks of age. F: Mandibular incisor morphologies of offspring of control and diabetic dams at three weeks of age; the incisors of offspring of diabetic dams were smaller and chalky white (arrow). G: Immunostaining of ameloblastin (AMBN) and amelogenin (AMGN) in the incisors of offspring of control and diabetic dams at three weeks of age.

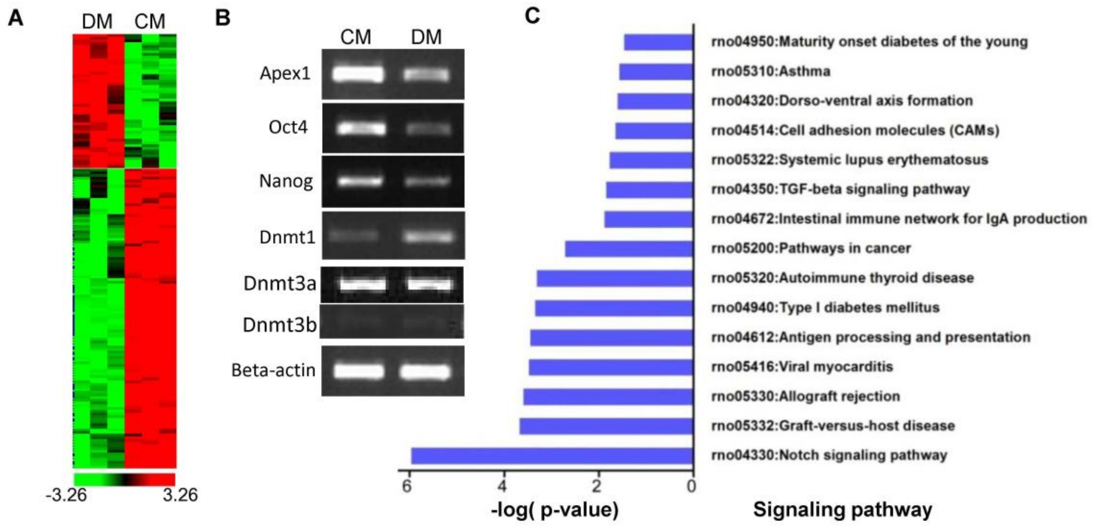

Supplementary Fig. S2 Maternal diabetes alters gene expression profiles in the cervical loop dental epithelium of offspring.

A: Heat map of differentially expressed genes in the incisor cervical loops of offspring of control and diabetic mothers (DM: offspring from diabetic mothers; CM: offspring from control mothers). B: Reverse transcription polymerase chain reaction analysis showed that *Apex1*, *Oct4*, and *Nanog* were upregulated, *Dnmt1* was downregulated, *Dnmt3a* expression was not changed, and *Dnmt3b* was not detected. C: Gene ontology analysis revealed that differentially expressed genes participate in various signalling pathways.
